# L'Hopital De La Charite De Paris
*For the material on which this account is based we are indebted to the monograph of M. Fernand Gillet, published in 1900, concerning L'Hôpital de la Charité, of which he was the director. This historical study was the outcome of much research among ancient documents, many of which are reproduced in full in the monograph, which is also illustrated.


**Published:** 1913-01-11

**Authors:** 


### L'HOPITAL DE LA CHARITE DE PARIS.*

### I.—THE ORDER OF BROTHERS OF SAINT-JOHN-OF-GOD.

On March 8, 1495, John Cieudad was born at Monte-Majore-el-Novo, in Portugal. At an early age he determined to devote his life to the relief of the sick poor. His earlier efforts were made at Grenada, where at first he worked alone, but in 1540 he determined to hire a house in which to receive the sick, and to that end collected alms. Eventually he opened his establishment, and his success was such that it was not long before the Bishop and townsfolk of Grenada bought a much larger building and presented it to him as a hospital. He died on March 8, 1550, aged fifty-five, but his work lived after him, and he left behind many disciples, who presently founded a congregation, which was approved in 1572 by Pius V., and in 1617 was made into a religious order by Paul V., known as that of the "Fréres hospitaliers de la Charité."

### The Beginnings of the Order.

It was under the nickname of their founder, "John of God," that his disciples made themselves into a society to help the poor. The order founded its first establishments at Cordova, Madrid, and Lucennes. To maintain among themselves a spirit of obedience and uniformity of government in their hospitals, the brothers elected a superior-general from among their numbers, whom they called "Major," while each hospital had a superior. In 1587 they possessed twenty-two houses, and Pope Sixtus V. confirmed the congregation by two briefs of June 10, 1585, and October 1, 1586, uniting all the hospitals into a single body, giving it the power of holding general chapters, of electing a general, provincials, and other superiors, and of drawing up a constitution. The first general of the order, Father Peter Soriano, a Spaniard, was elected June 27, 1587, at a chapter held in L'Hôpital de Saint-Jean-Calibite. In 1608 the brothers divided the order into two sections, each of which followed the same rules, but one devoting its services to Italy, the other to Spain.

A father-general was elected by each section. The Italian congregation was divided up into nine provinces, which by the middle of the eighteenth century together contained 163 convents and hospitals, with 8,662 beds and 150,000 sick under treatment. The Spanish congregation administered seven provinces, which at the same date contained 134 convents and hospitals with 4,028 beds and 46,345 patients receiving succour. In addition to the father-general, who directed each congregation, a provincial was appointed to look after the establishments in the several provinces, while a prior carried out the duties of a director for each hospital. The province of Saint-Jean-Baptiste de France comprised thirty hospitals, together containing 3,221 beds. The principal establishment, and the only one in which novices were trained in the French province, was L'Hôpital Saint-Jean-Baptiste de la Charité de Paris, which was the headquarters of the Father-Provincial. The other establishments dependent on it were situated at Cadillac, Moulins, Poitiers, Niort, La Rochelle, Effiat, Roye, Charenton, Vezins, Pontorson, les Convalescents de Paris, Château-Thierry, Condom, Saintes, Grenoble, Vizille, Celles, Avon-lès-Fontainbleau, Senlis, Romans, Ile de Ré, Vitry-le-François, Metz, Brest, Gayette, Clermont-Ferrand, Granville, Gondreville, and Alan-en-Comminge.

### II. L'HOPITAL DE LA CHARITE.

It was to this order that Marie de Médecis, wife of Henry IV., had recourse when she wished to put into execution her project for the establishment of a hospital. Up to this time the only institution of the kind open to the sick poor was the Hôtel Dieu, whose resources were taxed to the utmost. In 1601 the Queen sent to Florence for four religious of the order, and accordingly Father Bonelli and three brothers were despatched by the General to open the new hospital under the title of "Saint-Jean-Baptiste de la Charité," presently abbreviated to "les Frères de la Charité," and finally to "La Charité." The original building chosen by the Queen was a house situated "in the rue de Petite-Seyne, in front of the Porte de Malaquest, in the place occupied later by les Petits-Augustins." The first patients were received in 1602, and letters-patent of that year granted by Henry IV., empower the congregation to reside in France and to construct and erect hospitals. The new foundation was however not to remain long in the place originally chosen, for in 1606 Marguerite de Valois, wishing to establish on this site the convent des Petits-Augustins, entered into negotiations with the brothers of the order, which resulted in their exchanging it for another house situated at the corner of the rue des Saints-Pères and the rue Taranne, and since expropriated when the boulevard Saint-Germain was constructed. At that time it was the Hôtel de Sansac, and around it have sprung up the buildings, which from time to time have been added, and which together make up the hospital of to-day. In 1613 these buildings extended from the rue Taranne to the rue Jacob. The first erections were made at the angle of the boulevard Saint-Germain and the rue "Saint-Pierre," a name which was speedily corrupted to "rue des Saints-Pères." This last derived its name from an old chapel which had originally been the parish church of the Faubourg, dedicated to Saint Peter, and now handed over to the use of the brotherhood. In 1611 the chapel was ceded in perpetuity to the order by the curé and churchwardens of Saint-Sulpice.

### The Mother-House of the Order.

In 1613 the old church was pulled down, and the foundation-stone of a new and much larger one laid by Queen Marguerite de Valois, and dedicated to St. John the Baptist. It was solemnly inaugurated in 1621. The ground offered by the Queen was not of very great extent, and it was not long before an increase in the number of patients demanding admission necessitated an enlargement of the hospital. To this end in 1637 the brotherhood purchased further land lying between the rues Jacob, Saint-Benoît, Taranne and Saint-Pierre. Upon this, new, larger, and better ventilated wards were built. When completed the brothers desired to make the establishment the centre and mother-house of the order, and in this they were justified in that it was entirely under their control both in civil administration and in medical matters. It was within its walls that novices were taught the elements of medicine, surgery, pharmacy, and chemis-

* For the material on which this account is based we are indebted to the monograph of M. Fernand Gillet, published in 1900, concerning L'Hôpital de la Charité, of which he was the director. This historical study was the outcome of much research among ancient documents, many of which are reproduced in full in the monograph, which is also illustrated.

try, and before being admitted as members of the order, they were obliged to acquire the diploma of physician or surgeon, and to have a good acquaintance with the sciences connected with the art of healing. At first the beds in the wards were few, and it was not until 1742 that their number increased greatly. In this year there were 150 beds, fifty being in each of the three wards. Only men and boys were admitted into the hospital, and only patients suffering from curable diseases. Infectious and venereal maladies were rigorously excluded. A notable feature of the institution was that each patient had a bed to himself, a state of things which was uncommon in hospitals at that time, as may be seen from contemporary prints which often show several patients being treated in a single large bed. During the summer months a fourth ward was fitted up for the reception of patients desirous of being cut for stone. Alletz, writing in 1769, states that at that time there were fifty brothers and novices residing in hospitals.

They lived entirely by alms, and possessed nothing of their own. Patients were admitted for treatment and provided with everything. Necessity was the only passport recognised. Those who endowed beds had the right to name the persons whom they desired to occupy them, but otherwise admission was without any restrictions of class, race, or creed. The medical staff then consisted of two physicians, four brother apothecaries, two sworn surgeons, two brothers and five pupils. In addition there were ten surgeon apprentices, among whom some were earning their mastership by five years' gratuitous service. By 1788—a period when the hospital was at the height of its prosperity—it contained six wards and 208 beds. A bed could then be endowed for 10,000 livres. There were at that time 102 helpers in the hospital, an average of one for every two patients. This number was the result of the hospital being the headquarters and only seminary of the thirty-two houses possessed by the order in France. It must also be remembered that in addition to staffing the hospital the assistants prepared the drugs and medicines for the provincial establishments. The wards for the patients were all situated on the first floor, or built on archways on the side of the rue Taranne. On the ground floor were the kitchens, the refectory, the wash-house, the clothes-room, the administrative offices, the dispensary and the place reserved for the study of anatomy.

The second floor was occupied by the brothers, novices, and servants. It also gave space for meeting-rooms, and for the sick-room of the religious. No ordinary patient was accommodated there. During the eighteenth century the hospital was cited on all sides as the model of what such an institution should be from the point of view of medical skill, cleanliness, and hygiene. It was an absolute rule that every patient should be treated in a separate bed. This alone would explain the enviable notoriety the hospital enjoyed for a low mortality rate. Moreover, it had a great reputation for the treatment of lead-poisoning, and workmen attacked by lead colic flocked to it. Its principal entrance at that time was in the rue des Saints-Pères, near the chapel, which is now occupied by the Academy of Medicine, and facing the rue Perronnet. A passage existed also in the rue Jacob, and a third entrance led from the rue Taranne near a fountain, placed almost in the angle of the wall and on which could be read the inscription :—

*Quem pietas aperit miserorum in commoda fontem,*

*Instar aquæ, larga fondere monstrat opes.*

The brothers had among them a number of surgical celebrities. Two, Frère Jacques and Frère Cosme, were famous in their day as lithotomists. It was at this hospital that Mareschal, first surgeon to Louis XIV., undertook the operations which his contemporaries considered marvellous, and which laid the foundation of his success as a surgeon to royalty. Desault, a man of European reputation in his time, was successively surgeon-major to la Charité, and chief surgeon to the Hôtel-Dieu. Arrested during the Revolution and cast into prison, he was set free three days later owing to the complaints of the patients and pupils from whom he had been snatched. Two convalescent homes were possessed by the brotherhood, one situated in the rue du Bac, founded by Mme. de Bullion in 1650, and the other at Petit-Montrouge. Both were reserved for patients discharged from la Charité, but still requiring some minor treatment before returning to their ordinary life. The order drew revenues amounting at one time to 179,599 livres from houses, farms, lands, and other properties, as well as from fixed alms given by the King, the Duc d'Orléans, the chancellor, the farmers-general, and the "Comédiens Français," in addition to bequests and gifts and money received from the sale of goods and produce. Novices would appear to have paid for their accommodation, since no less a sum than 3,768 livres figures under the heading "pension des novices." Besides these revenues the brotherhood enjoyed certain privileges, exemptions, and immunities granted it by letters patent of Louis XIII. and Louis XIV., which it continued to make use of until the Revolution, which entirely changed the conditions under which the hospital was managed, as well as its name, which now became "L'hôpice de l'Unité."

### The Old Hospitals Abolished.

On December 22, 1789, the National Assembly, by a decree authorised by the King, charged the administrations of the various departments with the inspection and improvement of the working of hospitals and charitable establishments. The independence of the ancient French hospitals was thus abolished. In 1791 a decree of the Directoire transferred the direction of the hospitals to a committee of five, the departmental surveillance being transferred to the Commune of Paris. Once again the authority was changed by the Convention in 1794, which replaced the executive power by twelve commissions, of which one was charged with the task of public relief. A year later the hospitals were taken charge of by the Minister of the Interior. Finally, in 1801, by a decree of the Consuls, the administration of the hospitals was handed over to a general council, assisted by an administrative commission of which the Prefects of the Seine and of the Police were *de jure* presidents.

(*To be continued.*)

